# 

**Published:** 1860-04

**Authors:** 


### THE DENTAL REGISTER OF THE WEST.

EDITED BY

J. TAFT AND GEO. WATT.

April, 1860.

MONTHLY.--THREE DOLLARS PER ANNUM IN ADVANCE.

CINCINNATI:
J. T. TOLAND, PUBLISHER AND PROPRIETOR.

J. TAFT, DENTIST, No. 56 WEST FOURTH STREET.

